# Effects of Ambient Particulate Matter (PM_2.5_) Exposure on Calorie Intake and Appetite of Outdoor Workers

**DOI:** 10.3390/nu14224858

**Published:** 2022-11-17

**Authors:** Thavin Kumar Mathana Sundram, Eugenie Sin Sing Tan, Hwee San Lim, Farahnaz Amini, Normina Ahmad Bustami, Pui Yee Tan, Navedur Rehman, Yu Bin Ho, Chung Keat Tan

**Affiliations:** 1 Faculty of Medicine and Health Sciences, UCSI University, Kuala Lumpur 56000, Malaysia; 2 Cardiac Vascular Sentral, Kuala Lumpur 50470, Malaysia; 3 School of Physics, Universiti Sains Malaysia, Pulau Pinang 11800, Malaysia; 4 Faculty of Environment, University of Leeds, Leeds LS2 9JT, UK; 5 Scunthorpe General Hospital, Yorkshire and Humber, Scunthorpe DN157BH, UK; 6 Faculty of Medicine and Health Sciences, Universiti Putra Malaysia, Serdang 43400, Malaysia

**Keywords:** air pollution, particulate matter (PM_2.5_), Simplified Nutritional Appetite Questionnaire (SNAQ), calorie intake, outdoor workers

## Abstract

Malaysia has been experiencing smoke-haze episodes almost annually for the past few decades. PM_2.5_ is the main component in haze and causes harmful impacts on health due to its small aerodynamic size. This study aimed to explore the implications of PM_2.5_ exposure on the dietary intake of working individuals. Two phased 13-weeks follow-up study was conducted involving 440 participants, consisting of two cohorts of outdoor and indoor workers. Ambient PM_2.5_ concentrations were monitored using DustTrakTM DRX Aerosol Monitor. Data on Simplified Nutritional Appetite Questionnaire (SNAQ) and 24 h diet recall were collected weekly. The highest PM_2.5_ concentration of 122.90 ± 2.07 µg/m^3^ was recorded in August, and it vastly exceeded the standard value stipulated by US EPA and WHO. SNAQ scores and calorie intake were found to be significantly (*p* < 0.05) associated with changes in PM_2.5_ exposure of outdoor workers. Several moderate and positive correlations (R-value ranged from 0.4 to 0.6) were established between SNAQ scores, calorie intake and PM_2.5_ exposure. Overall findings suggested that long hours of PM_2.5_ exposure affect personal dietary intake, potentially increasing the risk of metabolic syndromes and other undesired health conditions. The current policy should be strengthened to safeguard the well-being of outdoor workers.

## 1. Introduction

Ambient air pollution significantly contributes to disease burden, especially heart and chronic respiratory diseases. This phenomenon leads to approximately 4.2 million annual premature deaths and is predicted to be the main contributor to global premature mortality by 2050 [1,2,3,4]. Higher exposure to air pollutants was observed in low- and middle-income countries [5,6,7]. Haze is a well-known air pollution phenomenon linked to persistent particulate matter episodes in Southeast Asia (SEA), significantly impacting the economy, health and environment [8]. Countries such as Malaysia, Singapore, Indonesia, and Thailand have been experiencing almost yearly smoke-haze episodes for decades due to the recurrent slash and burn agricultural activities and prolonged dry season [9,10]. Particulate matter with an aerodynamic diameter of less than 2.5 μm (PM_2.5_) poses a greater threat to human cardiovascular and respiratory systems due to its capability to reach the deepest recesses of lungs and penetration into bloodstream, impairing vital organs [11,12,13]. A recent animal toxicological study reported PM_2.5_ emitted during smoldering activity to be more toxic when compared to vehicular emissions at equal doses [14]. Prolonged hours of exposure to ambient PM_2.5_ was suggested to elicit more significant impacts on mortality and morbidity in the long run [15].

Malaysia’s prevalence of obesity has increased by four-fold since 1996 and inevitably has reached an epidemic level, especially for urban dwellers [16,17,18]. Now, Malaysia has the highest adult obesity rate among SEA countries. Obesity is the most preventable death globally and it utilizes substantial social resources [19]. The prevalence of obesity is expected to worsen by year 2025, given no implementation of control measures on its attributing risk factors [20,21]. Obesity is an outcome of complex interactions between intrinsic and extrinsic factors such as genetics, environmental, social and behavioral factors that eventually determine energy intake and expenditure [22]. Ultimately, the ratio of high energy intake to energy expenditure precedes the high rate of obesity [23]. A longitudinal study in China highlighted a 1.54% increase in average PM_2.5_ concentrations over the past 12 months to increase the body mass index (BMI), overweight and obesity by 0.27, 0.82% and 0.27%, respectively [24]. Additionally, similar observations have been reported in United States (US), Serbia, Italy, South Korea, Canada and Netherlands [25].

Several mechanisms have been proposed to explain the association between exposure to air pollution and unhealthy BMI. Air pollution can lead to disruption in metabolic pathways via increased inflammation in adipose tissue and oxidative stress, decreased efficiency in glucose metabolism and hepatic accumulation [26,27,28]. Prolonged exposure to air pollution was associated with neuroinflammation in the brain and thereafter, potentially altering appetite and eliciting anxiety-induced overeating behaviors [29,30]. Epidemiological findings indicated that airborne particles could induce pro-inflammatory responses in the central nervous system of children, resulting in poor appetite control, increased caloric intake and changes in basal metabolism [31]. Additionally, air pollution can negatively impact intentional efforts to lose weight by retaining behaviour for high-calorie intake [32]. Air pollution indirectly promotes a sedentary lifestyle by decreasing lung function, impairing exercise performance and discouraging regular physical activities [33,34]. Poor outdoor air quality can further exacerbate the situation causing excessive sedentary behaviour [35]. 

Without a doubt, industrialization and urbanization will continue to surge in Asian countries and are expected to follow suit by air pollution and related health issues. Although the negative impacts of air pollution were well researched, its impact on food intake remains scanty and rare, especially in developing countries. Secondly, most previous studies were focused on occupational-associated air pollutants such as vehicle admissions, mining dusts, construction dusts, and waste treatment odors [36,37,38,39,40,41,42,43]. This study aimed to determine the impact of PM_2.5_ exposure on food intake among outdoor workers via a cohort longitudinal approach. Results are anticipated to provide insights on possible underlying causes for the high prevalence of obesity in Malaysia, allowing early intervention and mitigation plans. 

## 2. Materials and Methods

### 2.1. PM_2.5_ Measurements

Ambient PM_2.5_ concentration was measured using DustTrak^TM^ DRX Aerosol Monitor (Model 8520, TSI, Shoreview, MN, USA) [44,45,46]. DustTrak^TM^ DRX consisted of a photometer and an optical counter, the concentration of PM_2_._5_ was determined by illuminates a laser diode into a sample stream, and the reflected light corresponding to the amounts of PM was captured by photodetector. The photometric voltage output from the DustTrak^TM^ DRX was translated into real-time mass concentrations of PM_2.5_. The reading was recorded at 5 min interval. During real-time monitoring, the inlet of the sampler was placed about 1.2 to 1.5 m above ground. All DustTrak^TM^ DRX were installed without 1 km radius of each participant recruitment site. The recorded data was then cross verified with the readings from Department of Environment (DOE) Malaysia. 

### 2.2. Study Population

This is cohort study with two cycles conducted at different timeframes; first cycle was conducted from August to November 2016, while the second cycle was conducted from June to August 2017. Sampling duration coincided with Malaysia’s monsoon season and the equatorial SEA region’s dry season, which had higher chances for a haze episode [47]. A total of 440 participants aged 18 and above, regardless of gender were recruited among working adults in Kuala Lumpur, Malaysia. Participants with outdoor exposure of more than 12 average hours per day were categorized as outdoor workers cohort, while those who spent less than 5 average hours outdoors daily were categorized as indoor workers cohort, as defined in previous study (Tovalin et al., 2006). Individuals with irregular or uncategorisable working hours were excluded from this study. Additionally, pregnant women and participants who were undergoing medication, particularly for respiratory conditions, were excluded. This study was ethically cleared by University Malaya Medical Centre Ethics Committee (UMMC EC) (approval code 20165-2447). The research was conducted with full compliance to Declaration of Helsinki and the Malaysian Guidelines for Good Clinical Practice (Guideline, 2001). Participants who fulfilled the selection criteria and willing to give consented were recruited in this study. 

### 2.3. Dietary Intakes 

Throughout the 13 weeks of study, all participants were required to complete a weekly self-administrated questionnaire consisting of 3 sections: (1) Sociodemographic, (2) Simplified Nutrition Appetite Questionnaire (SNAQ), and (3) 24 h diet recall (24hDR) log. The Simplified Nutritional Appetite Questionnaire (SNAQ) is the simplified version of the Appetite, Hunger and Sensory Perception (AHSP) questionnaire which originally developed to assess the appetite among community-dwelling elderly population in Netherland [48]. SNAQ was shown to have equal reliability, specificity, and sensitivity as original questionnaire in predicting malnutrition in nonspecialized population [49]. The SNAQ composed of four items that assess appetite, satiety, taste of food and number of meals per day, respectively. Each item was provided with five options with the scale of 1 to 5. Total scoring of SNAQ ranged from 4 to 20. Lower scores indicate higher risk of weight loss. Cutoff point of ≤14 was suggested to predict malnutrition and involuntary weight loss [50]. 

Information on calorie intake was assessed using an adapted and an interactive pre-tested 24 h diet recalls (24hDR) method [51]. This method was previously validated in a Malaysia’s national survey program [52]. The 24hDR were taken for three non-consecutive days over the week, including two weekdays and one weekend. The diet recall was conducted in the greatest possible details, which include the estimated portion size, volume of food, types, brands, and cooking methods, with the aid of some common household measurement photographs as reference. All recall data were analysed using the Nutritionist Pro 6.0 (Axxya Systems-Nutritionist Pro, Stafford, TX, USA).

### 2.4. Statistical Analysis

Socio-demographics were presented as categorical data, expressed in frequency and percentage. All outcomes were analysed as continuous dependent variables, presented as mean ± SD for normally distributed data or median (interquartile range) for non-normally distributed data. The changes in dietary intakes from baseline to last follow-up visit were analyzed using a general linear model (GLM) for repeated measures. The within-subjects factor was defined as the sampling time point. Indoor/outdoor was tested as the between-subject effect. Adjustments were made for individual-varying covariates, including gender, age, ethnicity, and smoking habit. Levene and Box M tests assessed the homogeneity of the variance and covariance structure of the dependent variables. The sphericity test of the residual covariance matrix was assessed using Mauchly’s sphericity test. Pearson’s two-tailed correlation coefficients were calculated to investigate the relationships between continuous variables. Results were considered significant if *p* < 0.05 with 95% of confidence interval. Statistical analysis was performed using SPSS 26.0 (IBM Corp., New York, NY, USA) for macOS.

## 3. Results

### 3.1. PM_2.5_ Data

**Figure 1:**
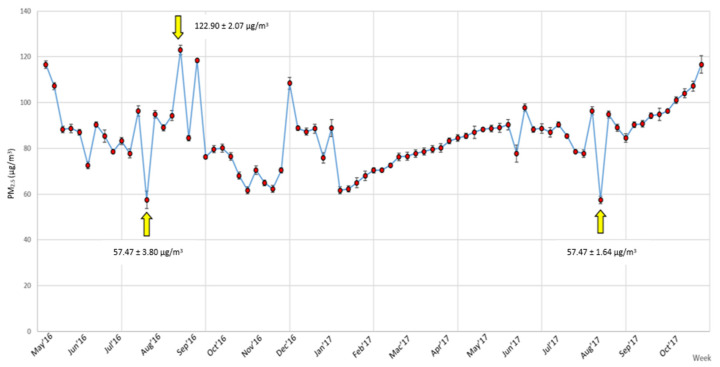
The PM_2.5_ concentration (µg/m^3^) changes in weekly basis during study period.

The changes in concentration of ambient PM_2.5_ on a weekly basis from May 2016 to October 2017 (80 weeks) is illustrated in Figure 1. The peak of ambient PM_2.5_ was charted at the third week of August, with maximum concentration of 122.90 ± 2.07 µg/m^3^. Minimum concentration of ambient PM_2.5_ were recorded twice at the fourth week of July 2016 and first week of August 2017, with respective concentration of 57.47 ± 3.80 µg/m^3^ and 57.47 ± 1.64 µg/m^3^. The average concentration of ambient PM_2.5_ recorded during the 80 weeks of assessment was 84.99 ± 1.69 µg/m^3^. 

### 3.2. Characteristics of Participant

Majority of the participants (*n* = 409, 92.9%) given their consent and completed the 13-week cohort study, while 7.1% of them (*n* = 31) dropped out due to the long follow-up and repetitive weekly assessments. Characteristics of participants are presented in Table 1. Majority of the outdoor workers are male (*n* = 201, 95.7%), while the majority of indoor workers are female (*n* = 124, 62.3%). The overall gender ratio was about two to one (male: female). About half of the participants aged 30 and above (*n* = 165, 41.3%); of which, 56.4% of the participants were indoor workers (*n* = 93), and 44.6% of them were outdoor workers (*n* = 72). Only a small portion of participants smoked (*n* = 52, 12.7%), and the majority of them (*n* = 47, 90.4%) came from the outdoor workers cohort.

### 3.3. Simplified Nutrition Appetite Questionnaire (SNAQ)

**Figure 2:**
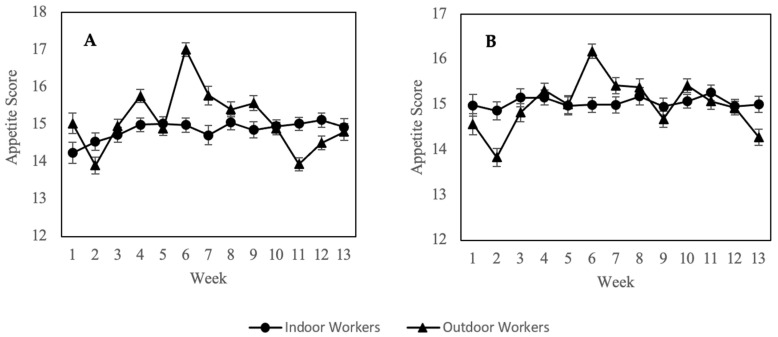
Change of mean appetite scores between indoor and outdoor workers over 13 weeks period in Cycle 1 (**A**) and Cycle 2 (**B**). Values are expressed as means ± standard error.

The changes in mean SNAQ scores of both cohorts during the 13 weeks of 2-cycle follow-ups are shown in Figure 2. Correlation analysis revealed a significant change (*p* < 0.05) in SNAQ scores among outdoor workers during the study period, which correlated moderately and positively with ambient PM_2.5_ concentration, with respective R-value of 0.541 and 0.453 corresponding to first and second cycle of assessment. No significant correlation was found on the changes of SNAQ scores among indoor workers (Table 2). 

### 3.4. Calorie Intake (24-Hour Diet Recall)

**Figure 3:**
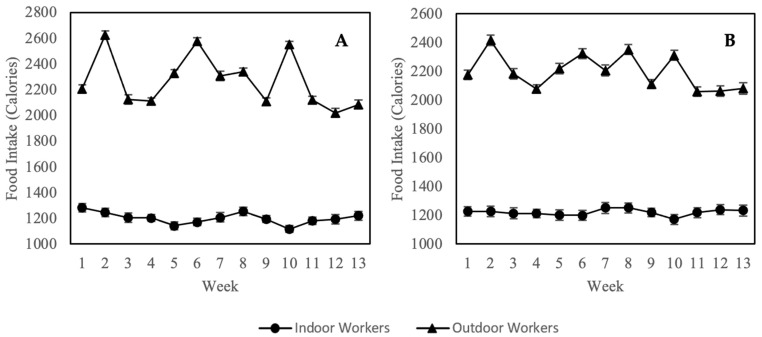
Change of calorie intake between indoor and outdoor workers over 13 weeks period in Cycle 1 (**A**) and Cycle 2 (**B**). Values were expressed as means ± standard error.

Changes in calorie intake of both indoor and outdoor worker cohorts are shown in Figure 3. The average calorie intake of outdoor workers was significantly higher (*p* < 0.05) than indoor workers for both cycles. When tested against changes in the ambient PM_2.5_ concentration, the changes in calorie intake among outdoor workers were found to be significant and moderately correlated, with R-value of 0.493 and 0.581 for cycle 1 and 2, respectively. No significant correlation was observed among indoor workers (Table 2). 

## 4. Discussion

Our findings revealed consistently high ambient PM_2.5_ throughout the study period. Our average recorded value was 84.99 ± 1.69 µg/m^3^, which is 2.4-fold more elevated than the healthy value of 35 μg/m^3^ as proposed by United States Environmental Protection Agency (US EPA) and 3.4 fold higher than 25 μg/m^3^ as proposed by World Health Organization (WHO) [53,54]. Haze affected most countries in the SEA region with at least one to two yearly haze episodes over the past decades [47]. In Malaysia, haze usually occurs during the dry season between June and September, with its most severe and prolonged haze recorded in September 2015 [55]. Similarly, this study observed the highest PM_2.5_ peak of 122.90 ± 2.07 µg/m^3^ during the third week of August. Countries within the equatorial region such as Indonesia experienced drought from June to September during its southwest monsoon, which provoked smoldering fire [56]. Agricultural activities such as anthropogenic drainage and peat harvesting reduced moisture content in the peat profile, rendering them more susceptible to smoldering [57]. Concurrently, the fierce southerly and south-westerly winds would intensify transboundary transport of air pollutants to the neighboring country, Malaysia, thus explaining soaring concentrations of PM_2.5_ in this study. On the other hand, the lowest PM_2.5_ peak recorded was attributed to frequent rainfalls. High relative humidity during the rainfall season effectively reduced ambient PM_2.5_ [58]. 

Amidst Asian countries, Malaysia’s prevalence of obesity was unbeatable highest due to their unhealthy eating habits [59,60]. The prevailing model of obesity is characterized by increased calorie intake and a sedentary lifestyle resulting in positive energy balance and excess fat storage [61]. Consumption of fast food and lack of physical activities for urban dwellers are known to be driving factors for obesity, but emerging evidence suggests other contributing factors for obesity [31,62,63]. Ambient air pollutant is a probable environmental obesogens associated with metabolic disorders such as insulin resistance, metabolic syndrome, and type 2 diabetes [64,65,66]. In this study, appetite was significantly (*p* < 0.05) associated with PM_2.5_ exposure of outdoor workers, with a moderate and positive correlation. Although its underlying mechanisms are poorly understood, appetite is known to be driven by Ghrelin hormone secretion, which may be disrupted by inhaling polluted air [67,68]. Ghrelin, an orexigenic hormone, is principally synthesized by oxyntic cells in the stomach and released in response to fasting [69]. High circulating plasma levels of Ghrelin act on the hypothalamus and vagus nerve to stimulate hunger, and its levels are rapidly downregulated after a meal [70]. A previous study showed that high PM_2.5_ exposure could drastically increase the recurrence of sleep disorders, indirectly increasing ghrelin secretion levels [71]. Chronic PM_2.5_ exposure also correlated with physiological stress, which has been implicated as a major contributor to ghrelin levels [72,73,74]. Notwithstanding, the moderate correlation found in this study suggests other contributing factors for appetite changes of outdoor workers, such as high energy expenditure [75], and changes in ambient temperature [76].

Besides appetite modification, air pollution is also suggested to elicit behavioral changes, particularly reducing the desire for exercise and increasing calorie intake [24]. Air pollution may induce various phycological distress such as anxiety and depression, thereby releasing cortisol hormone and increasing the appetite for high-energy foods [77]. Our findings reported that outdoor workers with prolonged ambient PM_2.5_ exposure underwent significant (*p* < 0.05) changes in their calorie intakes, which correlated positively with concentrations of PM_2.5_. Likewise, data from the China Health and Nutrition Survey (CHNS) involving 13,741 adult participants highlighted that urban residents with higher exposure to air pollutants were likely to have a higher intake of fat [78]. Toxicological analysis of urban ambient PM_2.5_ was linked to inflammatory response and oxidative stress through multiple pathways [79,80,81]. Elevated levels of pro-inflammatory factors such as interleukin-6 (IL-6) disrupt food intake regulation and exert a direct effect on weight gain [82]. Previous studies reported that Inflammation in the hypothalamus caused by PM_2.5_ is the primary cause of hyperphagia [83,84]. Such observation is worrisome because the synergistic effects of PM_2.5_ exposure and a high-fat diet can induce non-alcoholic fatty liver disease (NAFLD), a precursor for the manifestation of metabolic syndrome [85,86]. Additionally, this synergistic effect also induces glucose intolerance [87,88], intestinal damage [89], heart injury [90,91], atherosclerosis [92], and ankylosing spondylitis [93]. The low calorie intake observed among indoor workers is most likely due to the calorie restriction diet of most female workers who aims to lose weights. This concur with a previous study conducted in Malaysia reporting calorie restriction diet to be commonly practised by most female participants, including those with normal weight; they aim to achieve a better body image and satisfaction [94,95]. Additionally, stressful and depressive environments contribute to undereating or habit of meal skipping [96].

## 5. Conclusions

Haze is affecting most countries in SEA region, including Malaysia, with almost yearly sightings over past decades. PM_2.5_ is the main culprit causing adverse human health impacts due to its minuscule size. In this study, we charted the highest ambient PM_2.5_ peak of 122.90 ± 2.07 µg/m^3^ and mean ambient PM_2.5_ of 84.99 ± 1.69 µg/m^3^, which vastly exceeded the standard value stipulated by US EPA and WHO, as well as the average annual value in many developing countries. Significant positive and moderate correlations were reported between appetite, calorie intake and PM_2.5_ exposure of outdoor workers. Currently, the awareness of PM_2.5_ is immensely focused on its harmful impacts to the respiratory tract, while little is known about its effects on dietary intake. Thus, this study is anticipated to be the first large cohort study on this knowledge gap. Our findings suggested prolonged hours of PM_2.5_ exposure can alter appetite and increase calorie intake, potentially increasing the risk of metabolic syndromes and other undesired health conditions. The present work highlighted the importance of determining the health risk due to air pollution in Malaysia. Results from this study would be useful to guide the government, private sector, health professionals, non-governmental organizations (NGOs), and relevant stakeholders in strengthening existing regulations and enforcing preventive measures, particularly to protect outdoor workers.

## Figures and Tables

**Table 1:** Characteristics of Participants.

Characteristic	Indoor Workers	Outdoor Workers	Overall
**Gender (*n*/%)**			
Male	75 (37.7)	201 (95.7)	276 (67.5)
Female	124 (62.3)	9 (4.3)	133 (32.5)
**Age (years) (*n*/%)**			
≤30	106 (53.3)	138 (65.7)	244 (59.7)
31–40	62 (31.2)	49 (23.3)	111 (27.1)
41–50	13 (6.5)	22 (10.5)	35 (8.6)
>50	18 (9.0)	1 (0.5)	19 (4.6)
**Smoking Habit (*n*/%)**			
Yes	5 (2.5)	47 (22.4)	52 (12.7)
No	194 (97.5)	163 (77.6)	357 (87.3)

**Table 2:** Pearson Correlation Coefficient Matrix of the Measured Variables.

Variables		PM_2.5_ Concentration	
		Cycle 1	Cycle 2
**Appetite**	Indoor Workers	0.134	0.093
	Outdoor Workers	0.541 *	0.453 *
**Calorie Intake**	Indoor Workers	0.179	0.087
	Outdoor Workers	0.493 *	0.581 *

* Correlation is significant at the 0.05 level.

## Data Availability

Not applicable.
